# Early Contralateral Recurrence of Ovarian Endometriosis After Unilateral Surgery: A Case Report

**DOI:** 10.7759/cureus.95159

**Published:** 2025-10-22

**Authors:** Daniela Nakuci, Leart Berdica

**Affiliations:** 1 Department of Pathology, University Hospital of Obstetrics and Gynecology ‘Queen Geraldine’, Tirana, ALB; 2 Department of Pathology, Faculty of Medicine, University of Medicine, Tirana, ALB

**Keywords:** contralateral, endometrioma, laparoscopic surgery for endometriosis, periodic follow up, second ovary

## Abstract

Endometriosis is a gynecological condition that primarily affects women of reproductive age and is a leading cause of infertility. Ovarian endometriomas (OMAs) are cystic forms often seen in advanced stages of the disease. This report describes the case of a 26-year-old patient who developed a contralateral OMA three months after laparoscopic excision of a left OMA. She presented with lower abdominal pain, dysmenorrhea, and menstrual irregularities. Imaging confirmed a unilocular cyst in the right ovary, and laparoscopic removal verified an endometriotic cyst. This case highlights the potential for early recurrence and contralateral involvement in advanced endometriosis. These findings underscore the importance of careful postoperative monitoring and counseling, with attention to anatomical distribution, disease stage, age, and reproductive history to guide follow-up and management.

## Introduction

Endometriosis is one of the most prevalent benign gynecological disorders, histologically defined by the presence of functional endometrial glands and stroma located outside the uterine cavity. It was defined as a clinical entity for the first time in 1940 by Simpson [1]. Since then, a growing body of evidence has documented the clinical importance of endometriosis, and great interest by gynecologists, histopathologists, and surgeons has been shown in order to unravel its physiopathology and to develop the correct treatment protocols. Its prevalence is estimated to be approximately 10% to 20% among women of reproductive age and up to 50% among women with infertility [1, 2].

This chronic, estrogen-dependent disease is associated with a range of debilitating symptoms, including chronic pelvic pain, severe dysmenorrhea, dyspareunia, and infertility, all of which significantly impair a patient’s quality of life, daily functioning, and work productivity.

Clinically, endometriosis is broadly categorized into four main types based on the location and depth of ectopic lesions: superficial peritoneal endometriosis, ovarian endometriosis (commonly presenting as ovarian endometriomas (OMAs) or ‘chocolate cysts’), deep infiltrating endometriosis, which extends more than 5 mm beneath the peritoneal surface, and extrapelvic endometriosis involving distant sites such as the lungs, intestines, bladder, surgical scars, or other atypical locations. OMAs represent the most frequent manifestation of endometriosis, affecting 2%-10% of women of childbearing age and up to 50% of women with infertility. OMAs, in particular, are strongly associated with advanced-stage disease and pose a significant risk for recurrence and infertility, often necessitating careful long-term management and tailored surgical approaches [3, 4].

Jenkins et al. [5] were among the first to elucidate the anatomical dispersion patterns of endometriotic lesions. They found that there was an approximately 50% higher prevalence of ovarian endometriosis observed on the left side. A later report by Matalliotakis et al. suggested a two-fold rate of left endometriomas, as compared to endometriomas on the right. This was confirmed by other studies as well [6-7].

Even in our case, the patient had previously undergone a laparoscopic procedure for the excision of a deeply infiltrating OMA located in the left ovary. Laparoscopic ovarian cystectomy is widely considered an effective treatment option for women with OMA who present with severe symptoms or coexisting medical conditions that warrant surgical intervention [8].

Various factors, such as prior medical treatment, persistent pain, and the stage of the disease, particularly in cases classified as advanced (American Society for Reproductive Medicine (ASRM) stages III-IV), are known to significantly increase the risk of recurrence [9].

An unrandomized prospective study at two tertiary centers followed 366 patients who underwent laparoscopic excision of OMAs, with at least six months of postoperative or post-therapy follow-up. Advanced disease stage (P = .03) and prior endometriosis surgery (P = .003) were significant predictors [10].

This research article focuses on the recurrence of ovarian endometriosis within a year, particularly involving the contralateral ovary, and discusses it in the context of advanced-stage endometriosis. 

## Case presentation

A 26-year-old patient presented to our hospital for the second time with complaints of lower abdominal pain, dysmenorrhea, and menstrual cycle irregularities. She reported that these symptoms had persisted intermittently since her initial laparoscopic surgery for left ovarian endometriosis, performed three months prior, but had recently worsened.

On physical examination, the patient appeared pale, and abdominal palpation revealed significant tenderness and pain. Given her history and current symptoms, a pelvic ultrasonographic examination was performed, accompanied by a review of her surgical records.

**Figure 1:**
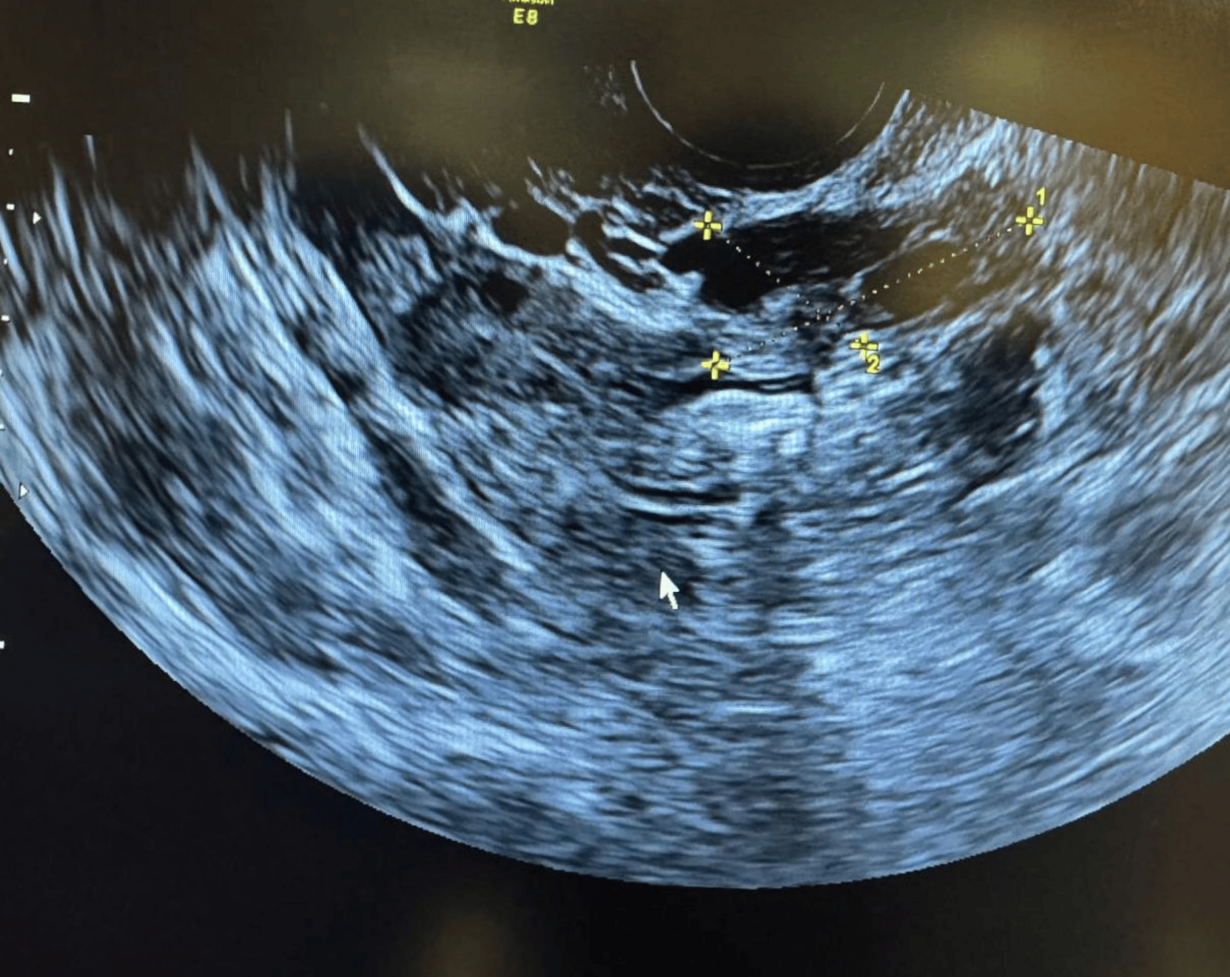
Ultrasonography of the left ovary after laparoscopic surgery

**Figure 2:**
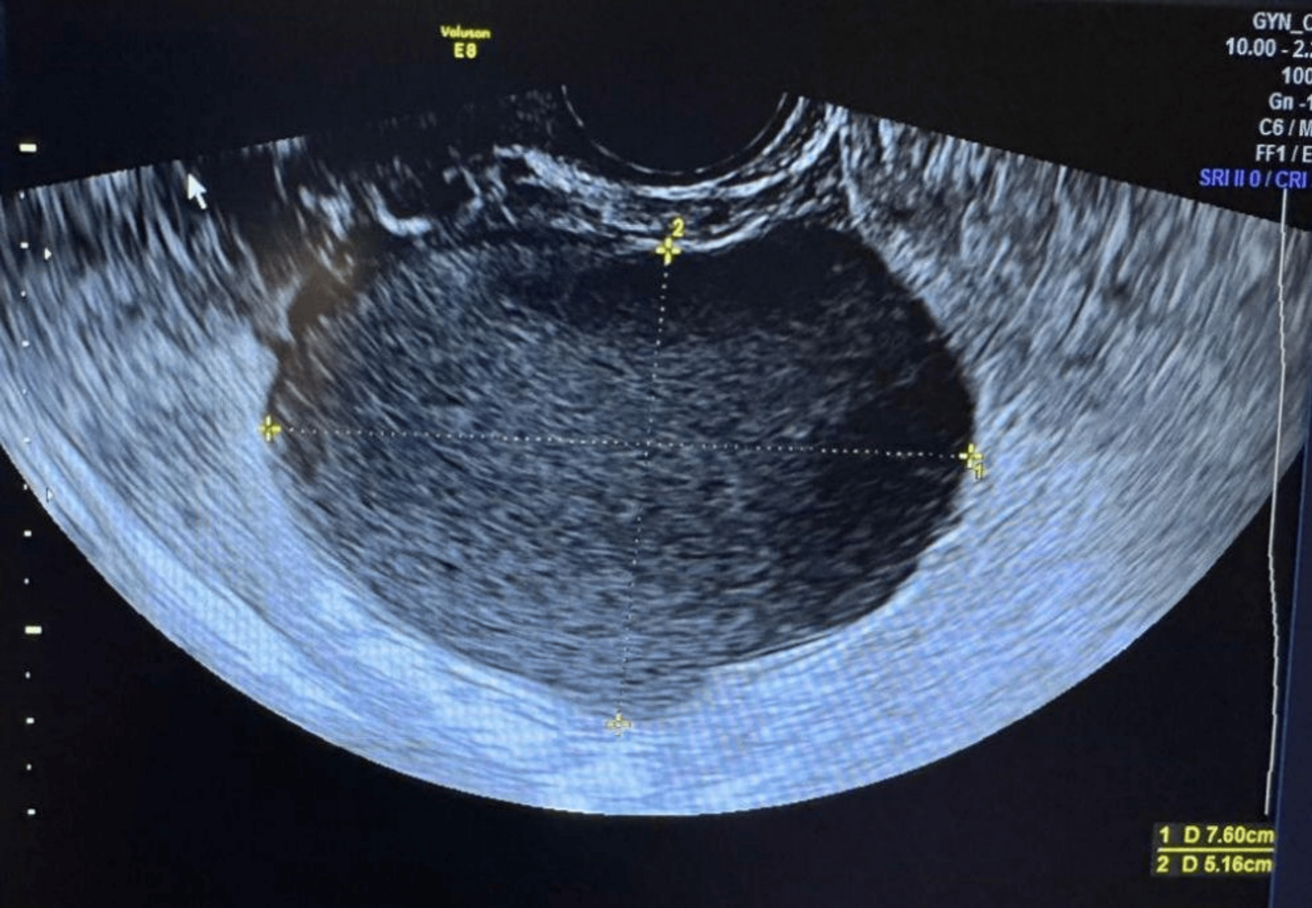
Image of the right ovary showing the presence of an endometrioma, measuring 7.6 cm × 5.16 cm (grayscale, transverse view).

Imaging demonstrated a normal left ovary three months post laparoscopic surgery (Figure 1). However, the right adnexa, which had previously appeared normal, now revealed a well-defined unilocular cystic lesion in the right ovary, measuring approximately 7.6 cm × 5.16 cm. The cyst exhibited homogeneous low-level internal echoes with the characteristic “ground glass appearance” typical of an OMA. No solid components, septation, or papillary projections were noted (Figure 2).

**Figure 3:**
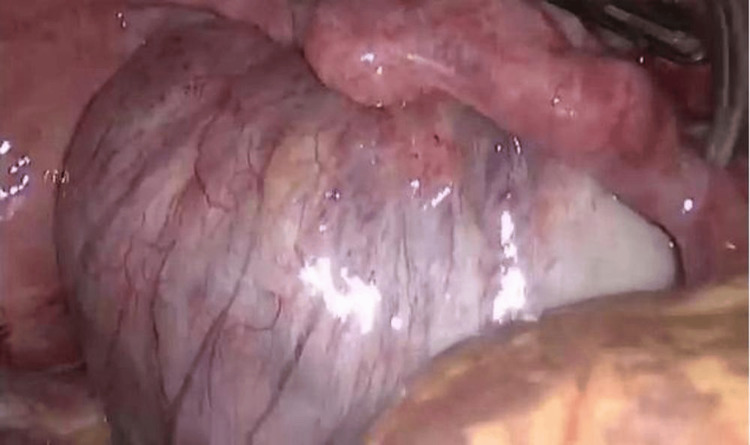
Laparoscopic view showing a large ovarian cyst with visible surface vasculature.

**Figure 4:**
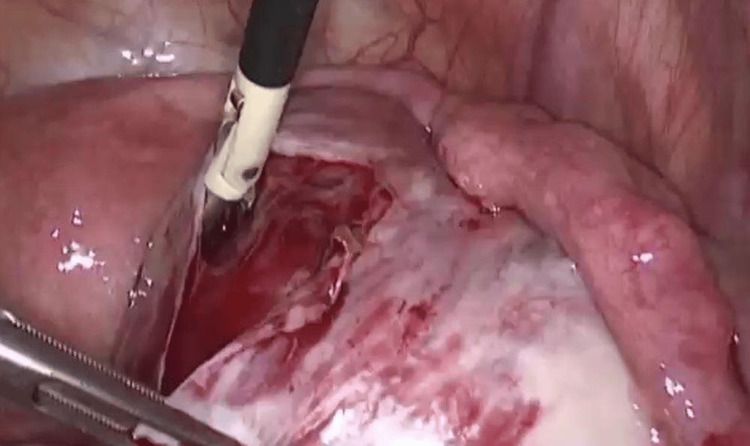
Intraoperative laparoscopic view of the cyst; removal of the cyst's capsule is shown.

A repeat laparoscopic excision of the right OMA was performed. Intraoperatively, the pelvis was inspected, and the right ovary was mobilized by releasing adhesions (Figure 3). A sharp cortical incision was made to access the cyst, a cleavage plane was identified, and the cyst capsule was completely stripped from the surrounding normal ovarian tissue (Figure 4).

**Figure 5:**
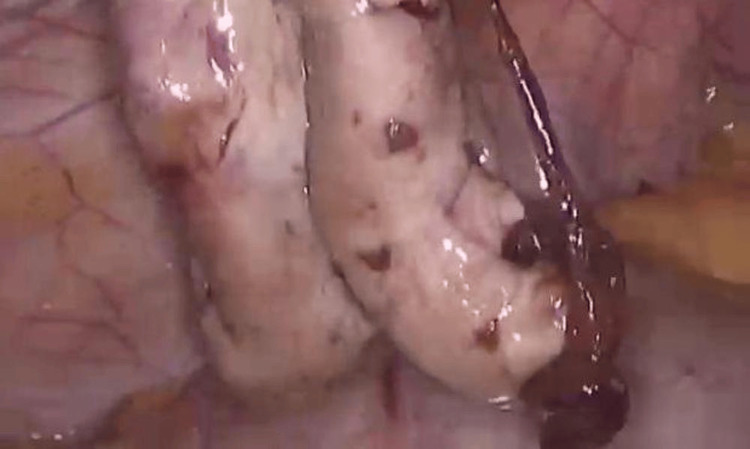
This image shows moments during the laparoscopic procedure: removal of the cyst capsule and its submission for histopathological examination.

The excised cyst capsule, containing areas of coagulated blood, was sent for histopathological examination (Figure 5).

Gross pathology showed an empty cystic wall measuring approximately 2 mm in thickness, with hemorrhagic areas and blood clots.

**Figure 6:**
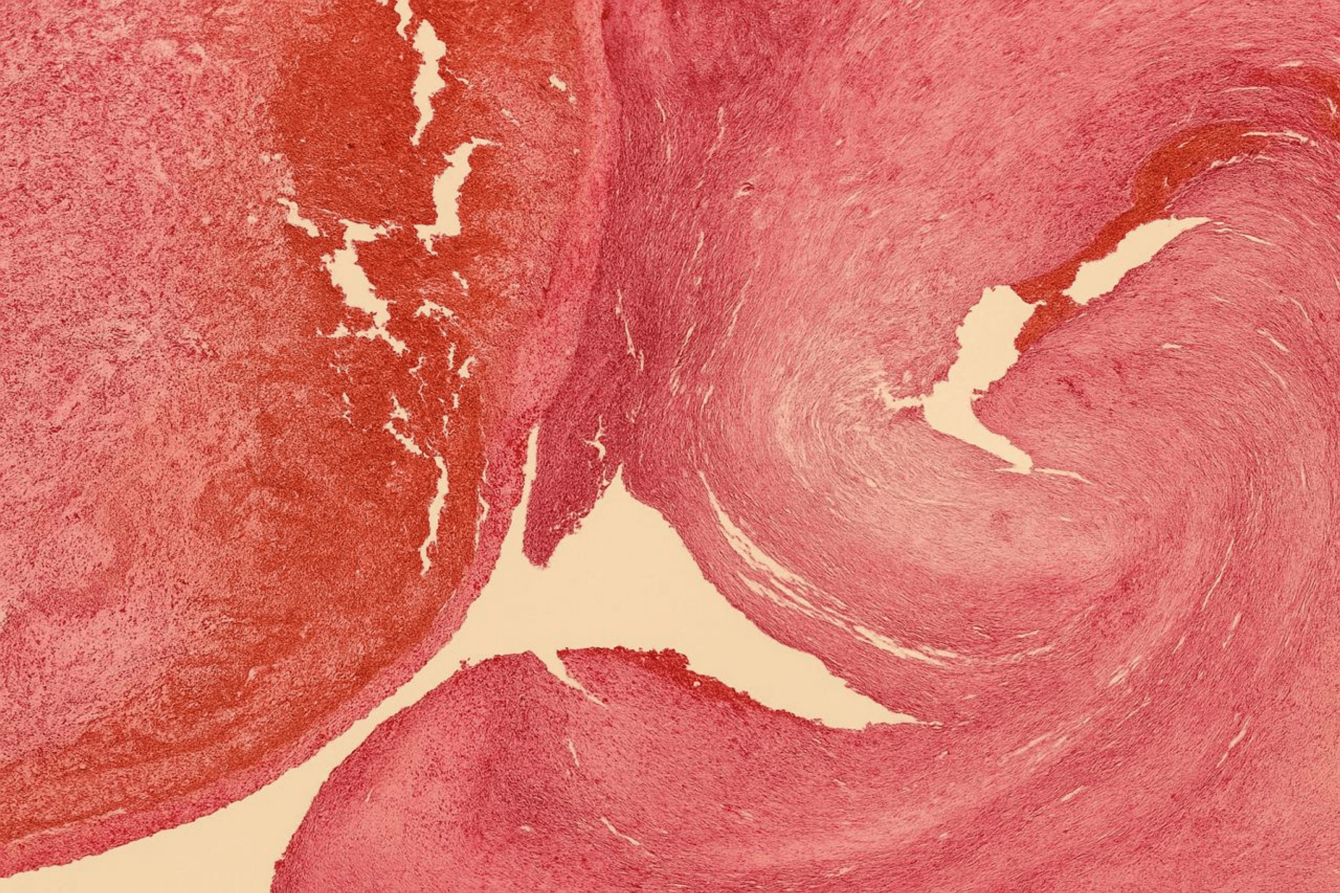
This histology image (H&E stain, 10x-low magnification) displays a thick fibrotic cyst wall with areas of hemorrhage, consistent with old blood.

**Figure 7:**
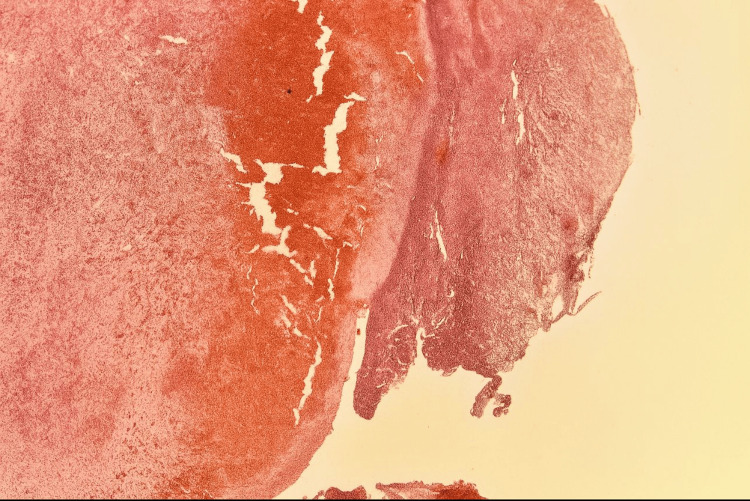
Histological image of an endometriotic cyst wall showing hemosiderin-laden macrophages and fibrous stroma. H&E stain, original magnification 40x.

Microscopically, the specimen demonstrated a single-layered epithelial lining with underlying endometrioid-type stroma exhibiting focal elastotic and fibrotic changes, as well as scant hemosiderin pigment (Figure 6). H&E stain at high power (40x objective) reveals endometrial stroma with hemorrhagic zones and hemosiderin-laden macrophages, typical of an endometriotic cyst wall (Figure 7).

These findings confirmed the diagnosis of a recurrent OMA in the contralateral ovary shortly after unilateral surgical treatment.

## Discussion

Ovarian endometriosis, particularly in the form of OMAs, presents a significant risk of recurrence despite surgical intervention, especially in cases of advanced-stage disease. Numerous studies have documented that recurrence can occur within the first year postoperatively, with rates increasing progressively over time.

For instance, Ghezi et al. (2001) [11] reported a recurrence rate of 17.3% in women with ovarian endometriosis, while other studies have demonstrated that a six-month course of hormonal suppression or dietary therapy after laparoscopic cystectomy does not significantly reduce recurrence compared with surgery alone. These findings suggest that complete laparoscopic excision of endometriotic tissue may be sufficient for effective management without the need for postoperative therapy [11-12].

Anatomical factors also appear to influence recurrence risk. Laterality, particularly left ovarian involvement, has been highlighted as a notable risk factor, with one study demonstrating a 3.7-fold increased likelihood of recurrence when the left ovary is affected [13]. This may be related to anatomical or physiological differences between the ovaries, although the underlying mechanisms remain unclear. While the time to recurrence does not appear to be strongly dependent on the initial lesion subtype, whether superficial peritoneal, ovarian endometrioma, or deeply infiltrating endometriosis, patients with severe or advanced-stage disease at the time of second-line surgery tend to experience higher recurrence rates [14].

Additionally, there is evidence suggesting that lesion severity can progress over time, with patients developing more aggressive subtypes even if the initial presentation was less severe.

Several factors have been implicated in influencing recurrence risk. The surgical approach and disease stage at initial treatment appear consistent across groups experiencing recurrence, underscoring the need for optimized surgical techniques and comprehensive excision [15].

Pathological features such as the depth of endometrial tissue infiltration into the ovarian cyst wall have also been identified as independent predictors of recurrence [16]. Biomarkers, including serum CA125 levels, along with cyst size, have been further associated with recurrence risk, although their utility in routine practice requires standardization [16].

Importantly, the definition of recurrence varies across studies, encompassing patient-reported symptoms such as pelvic pain, as well as clinical and imaging-based findings like recurrent cysts, nodules, or masses. This heterogeneity complicates the comparison of outcomes and underscores the need for standardized diagnostic criteria for recurrence.

## Conclusions

The recurrence of ovarian endometriosis, particularly in advanced-stage disease, underscores the need for comprehensive surgical excision, appropriate postoperative medical therapy, and individualized patient counseling. Future research should focus on refining risk stratification and developing optimized long-term management strategies to reduce recurrence rates and improve patient outcomes.
